# Letter from Dr. T. M. McIntosh

**Published:** 1889-10

**Authors:** T. M. McIntosh

**Affiliations:** Thomasville, Ga.; New York


NEW YORK, July, 1889.

*Editor of Atlanta Medical and Surgical Journal:*

Truly we live in a progressive age! Some years ago I spent some months in the various hospitals and clinics here, when all surgery was done with the elaborate dressing and technique of Lister; diagnostic laparotomy was unknown, and to open the abdomen was regarded as a serious operation. During every operation there was the warm and cold spray used,—operators, wounds and assistants were all kept constantly in a cloud of carbolic acid spray, and layer after layer of antiseptic dressing, oil silk, etc., were used with most minute attention as to which particular one should be first applied. At each subsequent dressing, the air was to be filled with the spray, and none admitted to the wound except when thus sterilized—how all this is changed! It is nothing but irrigation and bichloride of mercury solution. Nothing must touch a wound except after it has been made antiseptic with the solution, and during the operation the wound is frequently washed with the warm solution. No attempt is made to exclude non-sterilized air; formerly this was the *desideratum.*

It appears that there are some contradictions in the theory and the practice of antiseptic surgery, and it seems to the writer that the brilliant results that have been achieved have come because of the perfect *cleanliness* that is needed to carry out the theory of antiseptic surgery.

The admission of air to a wound during an operation is now known to do no harm, and being admitted, there is doubt as to whether an antiseptic solution, strong enough to kill the bacteria, would not also injure the tissues too much for the most satisfactory healing.

The assistants in an ovariotomy the other day would take their handkerchiefs and wipe the perspiration from their own and operator's dripping faces (the sweat dripping on the patient at times), and without sterilizing their hands, place them on the wound, instruments, etc.—an assistant put an instrument in his mouth and then in a wound!

Were the hands, handkerchief and mouth sterilized? No; but they were clean; not surgically clean, in the sense that any living bacteria were not present, but actually, aesthetically and practically, clean. Clean the hands of every one; clean patient, towels, sponges, ligatures, sutures, instruments,—everything! Use all the precautions of an antiseptic operation, except only using *warm* water, and it seems that the results would be the same.

Remove all foreign matter from a wound, clots, etc., drain freely, sew carefully, cover thoroughly with plenty of absorbent cotton, to keep a warm and equable temperature of the wound, keep the parts at *rest* and in gentle apposition, and all is accomplished that can be for rapid healing.

It should be called aseptic and not antiseptic surgery; though I suppose the former is a sequence of the latter. But no matter whether it is asepsis, with only warm water, or antisepsis with bichloride solution, surgery is now a wonderfully exact and charmingly successful science.

The marvelous success of Mr. Tait in laparotomies, with only irrigation, and his advocacy of laparotomy as a diagnostic measure, has given a wonderful impetus to abdominal surgery. Verily, his advice is being followed here; for an abdominal cavity is opened as readily as the ordinary physician would cut a felon, and it seems they deem it of as little danger.

One young enthusiast said, and insisted, that an exploratory laparotomy was attendant with no more danger than was the parturient act. Surely nature's handiwork should no longer claim our respect, and works on physiology should all be regarded as pathology, or *vice versa*, for we no longer seem to know what is pathological and what is physiological. In two days I saw four laparotomies, and in but one was the condition diagnosed substantiated by the operation, or remedied by it either. This exception was an abdominal hernia, the result of a former laparotomy.

A prominent surgeon of the northwest very modestly said that it was his custom to do such operations *without* opening the abdominal cavity, for the peritoneum had no sustaining power, and he brought only the edges of the separated muscles and fascia together by suture. One would think this the least dangerous way to cure the hernia, but there is no danger (?) now when you cut in, take out and look at a human intestine!

A woman, two months delivered of a child was supposed to have some disease of the right tube. Laparotomy; nothing found but a little congestion in the ileo-cæcal region, and the vermiform appendix red and adherent to the intestines. Well, this was an useless little appendage any way and it was the season of grape seed, and we will cut it off, in doing which the outer coat of the intestine was lacerated and hard to be stitched. While this operation was being done, I remarked to a visitor, who had seen the master Turk operate: "Ten years from to-day the doctor would not do this operation." "No," he said, "there will be great advance in that line." He did not seem to realize that I meant it would be left to nature, which in this case would surely have established a cure. He meant that some better *operation* would be found. Another was thought to be floating kidney. There was found a tumor of the intestine (probably malignant) to cure which an operation, probably fatal, would have to be done. The patient had not been advised of this possible contingency, and she was sewed up and left alone.

An enlargement in the left inguinal region, thought to be a tumor, was cut down upon carefully to the peritoneum, and an aspirating needle then inserted and *pus* was found. The surgeon well exclaimed: "What is man?" One M. D. from Mississippi said: "In my country an aspirator would have been used *before* the knife." A visiting physician told me that a woman was sent to a surgeon by a practitioner to remove a tumor of some kind from the abdomen. The surgeon could discover nothing. The practitioner coming in at that time, and finding none, insisted that he *had felt* it, and it must be there. The finger in the abdominal cavity will decide—laparotomy! Nothing!

All of these cases got well, I believe; but such successes should not warrant opening the abdominal cavity without more carefully learning the history of cases and by comparative signs and symptoms coming to more exact diagnosis, and finding symptoms and conditions more imperatively calling for operative treatment

(and likely to be benefited by it) and more seriously threatening life.

It was assuring to hear a distinguished gynecologist and a bold and successful operator, in a clinical lecture, protest against this frequent, prompt and wholesale diagnostic laparotomy; "for," he said, "one has no more right to do it than to differentiate a pleurisy from a pneumonia by opening the chest cavity." This is a strong way of putting it, but it gives you the idea. No one, I dare say, will question that the many bold and brilliant laparotomists have added to the years of human life, but that is no reason why more care should not be used in forming a diagnosis and the exercise of a more discriminating judgment before a resort to the knife. Surgery is advancing wonderfully. It is the exact science in our profession. It is the attractive and alluring field to all of us. One should be happy with an unlimited amount of it to do, and do it boldly, carefully and conscientiously, with proper confidence in one's art and power, and proper regard for the danger to human life that accompanies the major operations.

T. M. McINTOSH.

*Thomasville, Ga.*

